# Molecular typing of canine parvovirus type 2 by VP2 gene sequencing and restriction fragment length polymorphism in affected dogs from Egypt

**DOI:** 10.3389/fmicb.2023.1254060

**Published:** 2023-12-07

**Authors:** Asmaa Magouz, Ismail El-Kon, Enrique Raya-Álvarez, Enas Khaled, Noura Alkhalefa, Alaa S. Alhegaili, Manal F. El-khadragy, Ahmad Agil, Ehab Kotb Elmahallawy

**Affiliations:** ^1^Department of Virology, Faculty of Veterinary Medicine, Kafrelsheikh University, Kafrelsheikh, Egypt; ^2^Department of Gynaecology, Obstetrics and Artificial Insemination, Faculty of Veterinary Medicine, Kafrelsheikh University, Kafrelsheikh, Egypt; ^3^Rheumatology Department, Hospital Universitario San Cecilio, Granada, Spain; ^4^Department of Medical Laboratory, College of Applied Medical Sciences, Prince Sattam bin Abdulaziz University, Alkharj, Saudi Arabia; ^5^Department of Biology, College of Science, Princess Nourah bint Abdulrahman University, Riyadh, Saudi Arabia; ^6^Department of Pharmacology, Biohealth Institute Granada (IBs Granada) and Neuroscience Institute, School of Medicine, University of Granada, Granada, Spain; ^7^Departamento de Sanidad Animal, Grupo de Investigación en Sanidad Animal y Zoonosis (GISAZ), Facultad de Veterinaria, Universidad de Córdoba, Córdoba, Spain; ^8^Department of Zoonoses, Faculty of Veterinary Medicine, Sohag University, Sohag, Egypt

**Keywords:** canine parvovirus, PCR, RFLP, VP2 gene, Egypt

## Abstract

**Introduction:**

Canine parvovirus-2 (CPV-2) is one of the most common infectious diseases in dogs characterized by severe gastroenteritis, vomiting, and bloody diarrhea. Little information is available about this topic in Egypt, particularly in the Delta region. This study reports the prevalence and molecular analysis of CPV-2 variants collected from El-Gharbia and Kafrelsheikh governorates in the Delta of Egypt.

**Methods:**

In this study, 320 rectal swabs were collected from infected domestic dogs from two districts in delta Egypt. The samples were investigated by rapid immunochromatographic test and polymerase chain reaction for detection the prevalence of CPV-2 variants. The genetic characterization was performed using restriction fragment length polymorphism (RFLP) analysis and partial VP2 gene sequence.

**Results and discussion:**

The viral antigen was detected in (264/320, 82.5%) of samples by IC test, while PCR was found more sensitive by detecting (272/320, 85%) positive samples. The RFLP technique using M*bo*II restriction enzyme was successfully used for the differentiation of CPV-2c antigenic variants from CPV-2a/2b strains. Interestingly, the molecular and phylogenetic analysis revealed that both CPV-2a and CPV-2c are circulating in the study area. Deduced amino acid sequence analysis showed changes at residue (N426E) and residue (T440A).: Our results indicated that CPV-2 is prevalent among dogs in Egypt, and therefore further molecular and epidemiological studies of CPV-2 are warranted.

## 1 Introduction

Canine parvovirus (CPV) is a highly infectious and fatal viral disease of canines since the late 1970s. Despite intensive vaccination, the virus is still a leading cause of acute gastroenteritis and canine mortality, especially among non-immunized young puppies (Liu et al., 2017). Dogs of all ages can get parvovirus, but puppies are more sensitive to the infection showing clinical signs within 3–8 days, in the form of fever, hemorrhagic gastroenteritis, vomiting, and bloody or watery diarrhea (Kim et al., 2017). Canine parvovirus is classified in the family of *Parvoviridae*, *Parvovirinae* subfamily, and *Protoparvovirus* genus (Timurkan et al., 2019). It is a naked DNA virus containing a linear, negative single-stranded genome of approximately 5.2 kb in length. The viral DNA is featured by two major open reading frames (ORFs). The first ORF includes/encodes two non-structural proteins (namely NS1 and NS2) that involve in virus multiplication, while the second one encodes the capsid proteins (VP1 and VP2) (Saei et al., 2017).

It seems that the virus was a host mutant from the feline panleukopenia virus in 1978 in the United States after point mutations in few nucleotide in the VP2 gene, and consequently, the virus spreads worldwide (Timurkan et al., 2019). It was first reported as CPV type-2 (CPV-2) to be differentiated from canine parvovirus type 1 (CPV-1) which is non-pathogenic to canine species (Yip et al., 2020). A few years after the emergence of the original strain, two antigenic variants of CPV-2 emerged and were classified as CPV-2a and CPV-2b and within few months they had become the predominant types worldwide (Buonavoglia et al., 2001). The VP2 is the main capsid protein expressing the main antigenic determinants, and amino acid mutations in this protein have important biological effects such as changes in the antigenic properties, host range and viral pathogenicity (Zhou et al., 2017). Typing CPV-2 variants is mainly based on changes in amino acids in VP2 protein residue 426 (Asn in CPV-2a and CPV-2b besides Glu in CPV-2c), though other specific amino acid alterations in VP2 residues have also been observed (Buonavoglia et al., 2001).

The CPV2a and CPV2b variants are both circulating throughout the world and can infect both dogs and cats, however, they exhibit a low virulence in cats. In 1990, the novel variants with one amino acid alteration (CPV2a297A) and (CPV2b297A) in the VP2 gene replaced CPV2a and CPV2b (Charoenkul et al., 2019). The amino acid substitution (Asp-426 Glu) in VP2 gene generated a new variant, known as CPV-2c, that infects several canine breeds (Lambe et al., 2016). This new variant initially emerged in Italy in 2000 but recently has spread to other countries (Timurkan et al., 2019). This amino acid change (D426E) of CPV-2c strain is caused by the change of (T → A) in the third codon position at nt 4,062 ± 4,066, creating a M*bo*II restriction site (GAAGA) which is unique to CPV-2c (Liu et al., 2017). Therefore, these mutants (types 2c) can be distinguished from the other antigenic types (2a and 2b) by enzyme digestion using M*bo*II enzyme (Timurkan et al., 2019). However, restriction fragment length polymorphism (RFLP) analysis is not capable of differentiating CPV-2b from CPV-2a, because both types are not digested by *Mbo*II (Buonavoglia et al., 2001). Diagnosis of CPV-2 based on clinical signs is misleading since many other pathogens can cause similar symptoms in dogs. Therefore, a clinical diagnosis should always be confirmed with laboratory tests (Sun et al., 2019). A number of molecular assays (gene amplification- based) have been applied for CPV-2 diagnosis, such as PCR, real-time PCR, loop-mediated isothermal amplification, multiplex PCR, and RFLP (AL-Hosary, 2018). In Egypt, the virus was initially recorded in 1982, in military police dogs (AL-Hosary, 2018). Since then, it has been circulating among dog population in Egypt. In 2012, the presence of CPV2b in Egypt was confirmed by sequence analysis and in 2014, serotypes 2b and 2c were detected among dog population (Amthal, 2014). Genetic characterization was used in 2018 to identify genotypes 2a and 2b, with special emphasis on numerous mutations in genotype 2b (Zaher et al., 2020). Another Egyptian study revealed the predominance of CPV-2c strains among the collected dog samples (Ndiana et al., 2022). At present, all variants (CPV-2a, CPV-2b and CPV-2c) are reported in Egypt (AL-Hosary, 2018; Soliman et al., 2018; El-Neshwy et al., 2019). Although several research studies regarding the molecular characterization of CPV variants in Egypt are present (AL-Hosary, 2018), for the best of our knowledge no previous reports were conducted on El-Gharbeya and Kafrelsheikh governorates in the Delta of Egypt Clearly, further studies are needed to highlight the different CPV-2 variants circulating in Egypt in Nile Delta region. The present study was designed to perform molecular characterization of CPV-2 variants from clinically infected dogs in the El-Gharbia and Kafrelsheikh governorates, using PCR and RFLP followed by VP2 sequencing.

## 2 Materials and methods

### 2.1 Ethical considerations

The study was performed according to the regulating rules of Institutional Review Board of Faculty of Veterinary Medicine, Kafrelsheikh University, Egypt (IRB number KFS-2019/09).

### 2.2 Study area and sampling

A total of 320 rectal swabs were collected from private veterinary clinics in El-Gharbia, and Kafrelsheikh governorates (Delta of Egypt) during the period from September 2019 to January 2021 from clinically infected domestic dogs showing fever, bloody diarrhea, dehydration and vomiting with history of previous vaccination in some dogs. The studied area, El-Gharbia and Kafrelsheikh governorates are based in the middle of the Delta of Egypt. The data of each puppy regarding age, breed, sex and clinical signs are recorded in Table 1. Samples were collected by licensed veterinarians following obtaining informed consent from the animals’ owners.

**Table 1:** Details of examined puppies (breed, age, sex, clinical signs and vaccination status).

Number of samples	Bread	Age (months)	Sex	Vomit/diarrhea	Vaccination status	PCR(−) samples
10	Griffon	3	Male	Severe	Not vaccinated	+
4	Husky	3.5	Female	Severe	Not vaccinated	−
8	Pit bull	3	Female	Moderate	Vaccinated	+
12	Pit bull	2	Male	Severe	Unknown	−
2	G. Shepherd^*^	3	Female	Mild	Vaccinated	+
6	Golden	6	Female	Moderate	Vaccinated	+
4	Pit bull	4	Male	Moderate	Vaccinated	+
6	G. Shepherd	4	Male	Mild	Vaccinated	+
8	G. Shepherd	2	Male	Severe	Unknown	+
4	Pit Bull	4	Male	Mild	Vaccinated	+
10	G. Shepherd	2.5	Female	Moderate	Not vaccinated	−
10	G. Shepherd	2.5	Male	Severe	Not vaccinated	+
12	G. Shepherd	2.5	Female	Severe	Vaccinated	−
10	Griffon	12	Female	Mild	Vaccinated	+
12	G. Shepherd	3	Female	Moderate	Not vaccinated	+
4	Pit Bull	6	Male	Moderate	Vaccinated	−
10	Golden	3.5	Male	Mild	Vaccinated	+
10	Pikinwa	12	Male	Mild	Vaccinated	+
8	Boxer	2	Male	Severe	Not vaccinated	−
10	Pit bull	3	Male	Moderate	Unknown	−
4	Pit Bull	2	Male	Severe	Vaccinated	+
6	Golden	2	Female	Severe	Vaccinated	+
8	Pit Bull	3	Female	Severe	Vaccinated	+
4	Pit Bull	2	Male	Moderate	Not vaccinated	+
2	G. Shepherd	2.5	Male	Severe	Vaccinated	−
10	G. Shepherd	2.5	Female	Severe	Vaccinated	+
14	G. Shepherd	3	Male	Severe	Vaccinated	+
4	Pit Bull	2.5	Male	Severe	Not vaccinated	+
6	Pikinwa	3	Male	Severe	Vaccinated	−
4	Pit Bull	3	Female	Severe	Not vaccinated	+
8	Pit Bull	6	Male	Moderate	Vaccinated	+
2	G. Shepherd	4	Male	Severe	Unknown	+
8	G. Shepherd	3	Female	Severe	Not vaccinated	−
10	Golden	12	Male	Moderate	Vaccinated	+
10	Golden	4	Male	Mild	Vaccinated	−
14	Boxer	3.5	Female	Severe	Unknown	+
8	Boxer	2	Male	Moderate	Not vaccinated	+
14	G. Shepherd	6	Male	Moderate	Not vaccinated	+
6	G. Shepherd	8	Female	Severe	Not vaccinated	+
16	Golden	6	Male	Severe	Unknown	−

G. Shepherd^*^ = German shepherd.

Rectal swabs were immersed in labeled tubes containing sterile phosphate-buffer saline (PBS) with 10% of antibiotic solution and centrifuged at 12.000 rpm (10 min). The supernatant fluids were then collected and kept at −80°C until processing.

### 2.3 Rapid Immunochromatic test for CPV antigen detection

All rectal swabs were tested by CPV rapid Ag detection kits (APETCARE) (China) as per manufacturer’s instructions. Briefly, fecal samples were collected from dogs using rectal swabs. The swabs were then introduced into the specimen tube containing 1 mL of assay diluent. The samples were mixed with the diluent and applied into the sample hole of the device of the test strip. Positive results are indicated by a visible T band in the corresponding testing window.

### 2.4 DNA extraction

The Viral genomic DNA was extracted from 300 μL of rectal swab suspensions using Gene Jet Viral DNA/RNA Extraction Kit (ThermoFisher Scientific, United States) as per the manufacturer’s protocol then the purified DNA was preserved at −20°C till used.

### 2.5 PCR amplification of VP2 gene

The PCR was conducted in 25 μL volumes, consisting of 12.5 μL of 2X PCR Master Mix (ThermoFisher Scientific, USA), 1 μL of forward and reverse primers, 5 μL of DNA, and 5.5 μL of PCR grade water. PCR was conducted in an Applied biosystem 2,720 thermal cycler using 2 sets of primers which amplify a 583 bp of the VP2 gene. DNA was amplified using primers described elsewhere (Buonavoglia et al., 2001) (Table 2). The PCR cycle condition comprised an initial denaturation step at 94°C for 5 min, followed by denaturation at 95°C for 30 s, annealing at 55°C /2 min and extension at 70°C/2 min (35 cycles), and a cycle of final extension at 72°C for 5 min. Regarding the positive control samples, DNA was extracted from a lyophilized Vanguard Plus CPV vaccine (Pfizer), while the negative control tube included only primers and nuclease free water to reach the final volume. Visualization of the resulting amplified PCR products was performed using 1.5% agarose gel electrophoresis and amplicons’ size was estimated using the 100 bp DNA marker (ThermoFisher Scientific, US). PCR -positive products were purified with purification kit (Thermo Fisher, United States) as per the manufacturer’s protocol.”

**Table 2:** Oligonucleotide primers used for amplification of VP2 gene of suspected CPV infected field cases.

Primer	Oligonucleotide sequence	Gene	Length of amplified fragment	Reference
Forward	5’ CAGGAAGATATCCAGAAGGA 3’	VP2 gene	583 bp	Buonavoglia et al. (2001)
Reverse	5’GGTGCTAGTTGATATGTAATAAACA 3			

### 2.6 Restriction fragment length polymorphism analysis

This step was carried out by digestion of purified amplicons with 5 units of the M*bo* II restriction enzyme (Fast Digest, ThermoFisher Scientific, USA) following the manufacturer’s instructions. Briefly, 10 μL of DNA, 1 μL of restriction Enzyme, 2 μL of green buffer and 17 μL of nuclease free water were mixed and incubated at 37°C in a heat block (5 min). Then, the enzyme was inactivated by heating at 65°C for 5 min. The cleavage manner of the amplicons was detected on 2% agarose gel.

### 2.7 DNA sequencing and phylogenetic analysis

QIAquick PCR product extraction kit (Qiagen, Valencia) was used to purify amplicons of the CPV-2 VP2 gene of four selected isolates based on different geographical areas. Using the same PCR primers, isolates were sequenced by BigDye Terminator V3.1 cycle sequencing kit using an Applied Biosystems 3,130 genetic analyzer (ABI, USA). The resulting sequence data were then submitted and deposited in the GenBank databases under the following accession numbers MW544032 (CPV/Egy1/2021), MW544033 (CPV/Egy2/2021), MW544034 (CPV/Egy3/2021), and MW544031 (CPV/Egy4/2021). The VP2 gene’s identity to GenBank accessions was established using blast analyses (BLASTn).^1^ By using the CLUSTAL W Multiple Sequence Alignment tool of the MEGA X software, the nucleotide sequences were then aligned and compared with other CPV reference strains accessible in the GenBank database, and then translated into amino acid sequences. MEGA X software was used for phylogenetic analyses using the neighbor-joining method and 1,000 bootstrap repetitions.

### 2.8 Sequence retrieval and alignment

**Figure 1:**
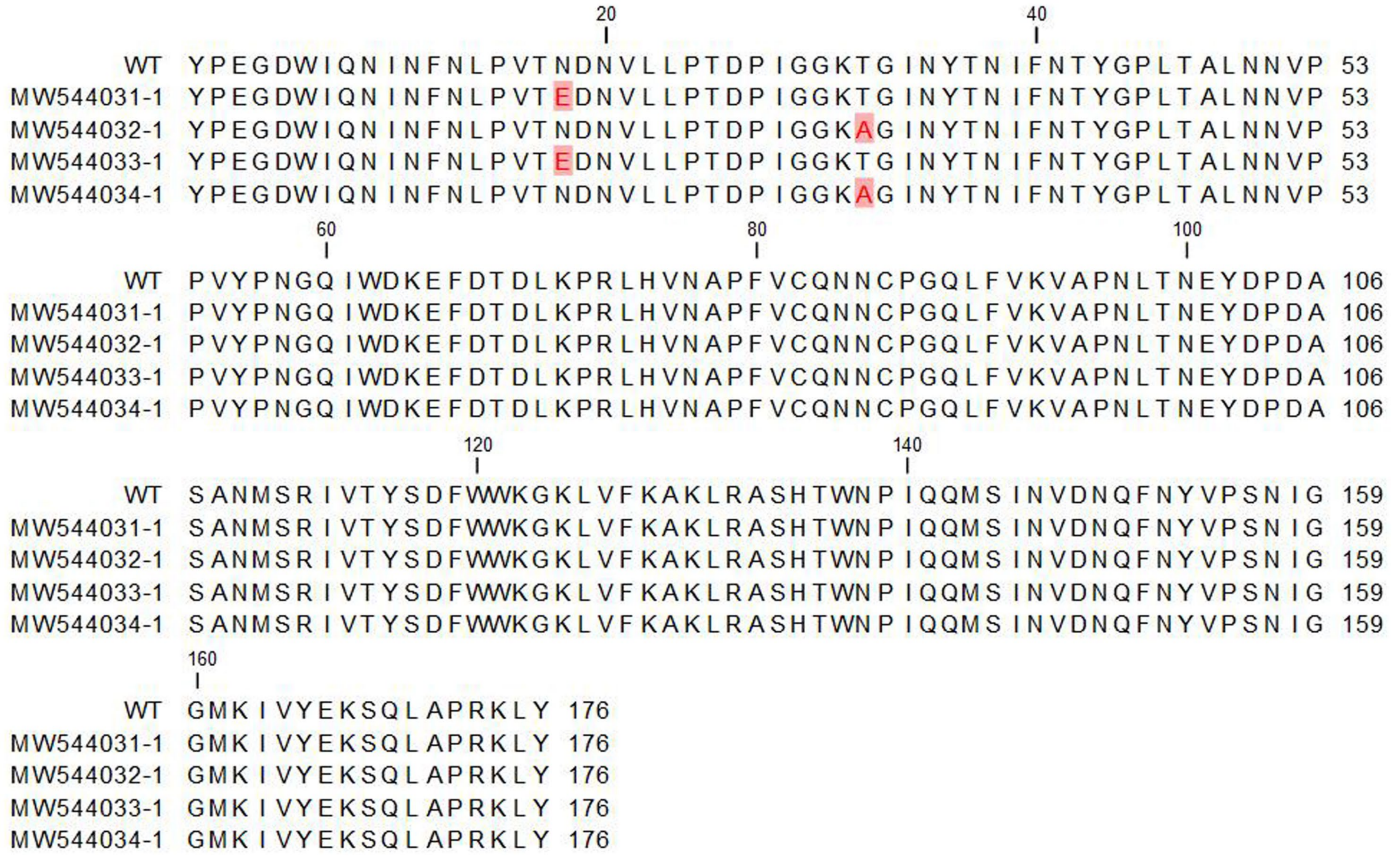
Multiple sequence alignment of the retrieved canine parvovirus VP2 proteins with the reference sequence (NP_955539.1). The alignment was performed by CLC genomic software.

In this study, sequence retrieval and alignment of canine parvovirus VP2 sequences were performed using the CLC Genomics Software. The primary objective was to align these sequences with the wild-type reference sequence (NP_955539.1). The alignment process facilitated the identification of conserved regions and potential mutations, shedding light on the genetic diversity and possible implications for virulence and vaccine development (Figure 1).

### 2.9 Molecular modelling

To determine the functional importance of the mutated residues in the retrieved sequences, requests for building 3D molecular models were submitted to the SWISSMODEL server (Waterhouse et al., 2018). The model building and quality were assessed by GMQE and QMEANDisCo Global score (Studer et al., 2020). To evaluate the influence of mutations on the collective binding potency amid interactions among VP2 protein monomers, computations were carried out to gauge both the binding efficacy and the dissociation constant (Kd). The analysis was performed at “Predicting the change in proteins binding affinity” tool (PANDA) (Abbasi et al., 2021).

### 2.10 Prediction of changes in canine capsid VP2 protein antigenicity

Molecular modeling investigations indicated that the predicted mutations are situated on the outer aspects of the capsid VP2 viral protein and are accessible to the surrounding liquid. This could potentially alter the immune recognition properties or antigenicity of the canine VP2 protein. Inquiries to assess the antigenicity of both the original and altered versions of the VP2 protein were submitted to the VaxiJen server (Doytchinova and Flower, 2007).

## 3 Results

### 3.1 Clinical manifestations and CPV antigen detection

In the present study, 320 clinically suspected CPV-2 infected puppies were clinically investigated in private veterinary clinics for pet animals in El-Gharbia and Kafrelsheikh provinces. The animals showed clinical signs ranging from mild to severe (Table 1). Out of 320 examined rectal swabs, 264/320 were found positive for CPV antigen by rapid IC test. The infection rate was higher in dogs aged less than 6 months (224/272) (82.3%) while dogs in the aged group of 6–12 months exhibit a lower infection rate (48/272) (17.6%). The German shepherd breed was found to be the most predisposed breed in this study (112/272) followed by Pit bull (72/272), Golden (40/272), Pikinwa (16/272) Boxer (24/272), Griffon (8/272).

### 3.2 PCR amplification of CPV-2 VP2 gene

Using conventional PCR, 85% (272/320) of samples were identified as positive by successful amplification a 583 bp of VP2 gene.

### 3.3 RFLP characterization for CPV strains

Restriction Fragment Length Polymorphism was performed successfully on 272 PCR-positive samples. The RFLP analysis of 272 samples classified 69 samples as CPV-2c strains by digestion of the purified DNA fragments into 500 bp and 83 bp fragments while the other 203 samples remained undigested indicating that they belong to CPV -2a/b strains.

### 3.4 Sequence alignment and phylogenetic analysis

**Figure 2:**
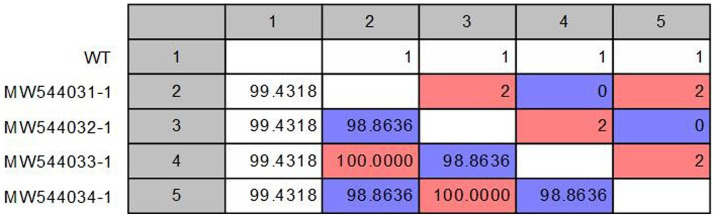
Pairwise comparison of the retrieved canine parvovirus VP2 proteins with the reference sequence (NP_955539.1). The upper right diagonal panel is the number of amino acid differences. The lower left diagonal panel is the identity%.

**Figure 3:**
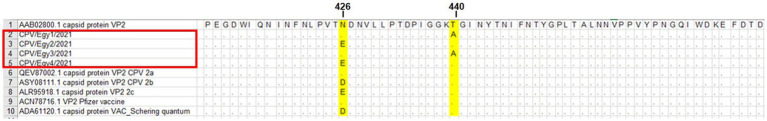
Deduced amino acid sequence alignment of CPV/Egy1/2021, CPV/Egy2/2021, CPV/Egy3/2021 and CPV/Egy4/2021 showing amino acid substitution at residue 426 and 440 in partial VP2 gene. Dots indicate identical letters.

**Figure 4:**
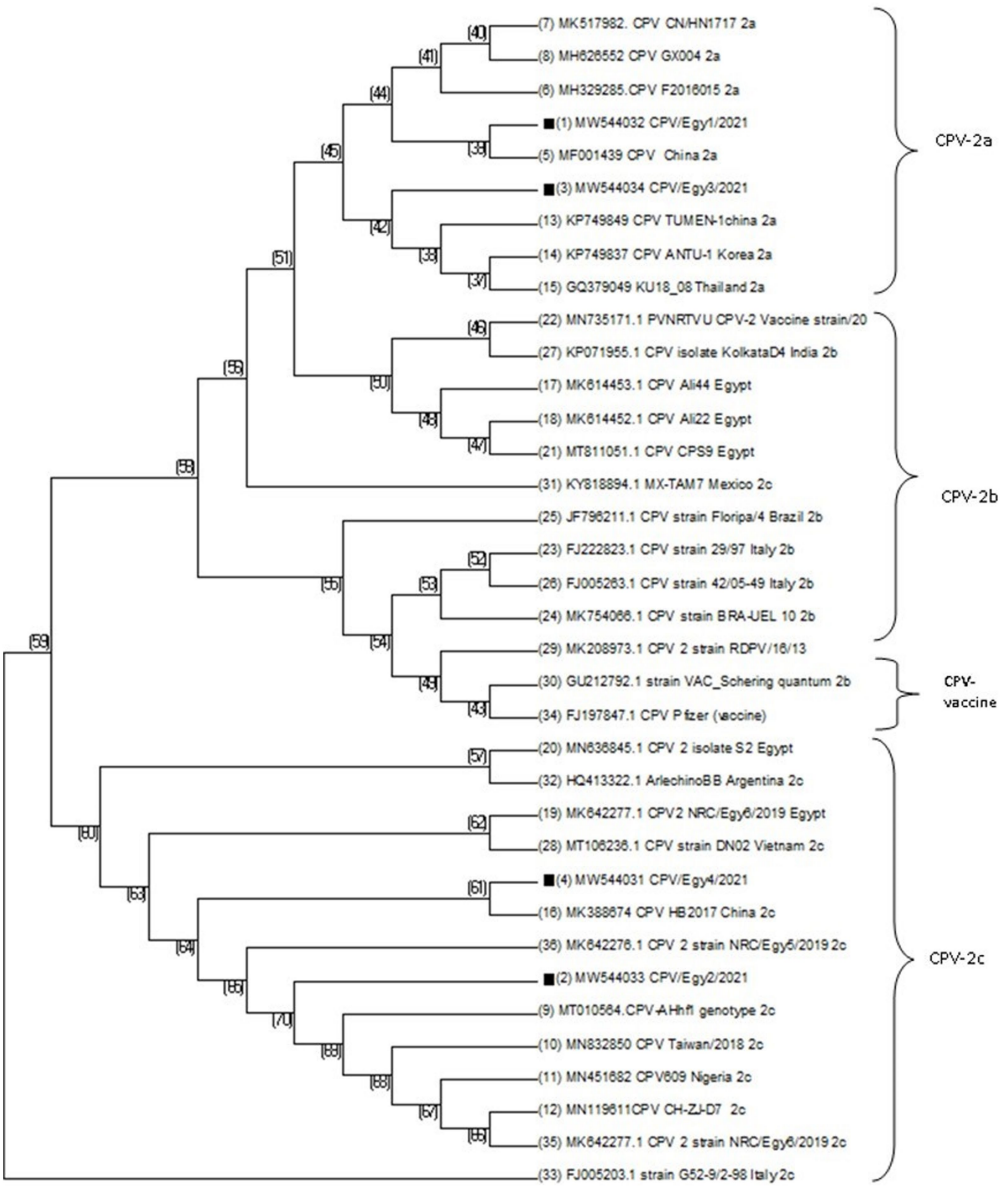
Phylogenetic analysis of CPV-2 samples based on VP2 nucleotide sequences of previously published sequences in Genbank database. Phylogenetic tree was constructed and prepared via multiple alignments of nucleotide sequences and analyzed using Neighbor-Joining method with bootstrapping (1000).

Pairwise statistical analysis of the sequences indicated that there were from 0 to 2 substitutions of amino acids, suggesting a sequence identity ranging from 99.43 to 100% (Figure 2). Analysis of the nucleotide and deduced amino acid sequences showed changes at position 1,278–1,280 resulting in (Asn → Glu) amino acid change at residue 426 (N426E) of the VP2 in strains CPV/Egy2/2021 and CPV/Egy4/2021 which is CPV-2c specific. Another amino acid substitution at residue 440 (Thr → Ala) (T440A) was observed in strains CPV/Egy1/2021 and CPV/Egy3/2021 compared to the reference strain (accession number AAB02800.1) (Figure 3). The Phylogenetic analysis showed that the resulting CPV VP2 partial sequences belonged to two different clades CPV-2a and CPV-2c. The strains CPV/Egy1/2021 and CPV/Egy3/2021 were clustered in the CPV-2a clade with 99.4–100% identity to other Egyptian and non-Egyptian (China, Korea, Thailand) strains. Whereas, strains CPV/Egy2/2021 and CPV/Egy4/2021 were clustered in CPV-2c clade with 99.6–100% identity to other Egyptian and non -Egyptian (Taiwan/Vietnam/China/Nigeria) strains. In addition, a comparison of the current 4 Egyptian CPV sequences to the commercially used vaccine strains CPV Pfizer (FJ197847.1) and VAC Schering quantum (GU212792.1) proved that the vaccinal strains were from a different branch (Figure 4).

### 3.5 Molecular modeling studies

**Figure 5:**
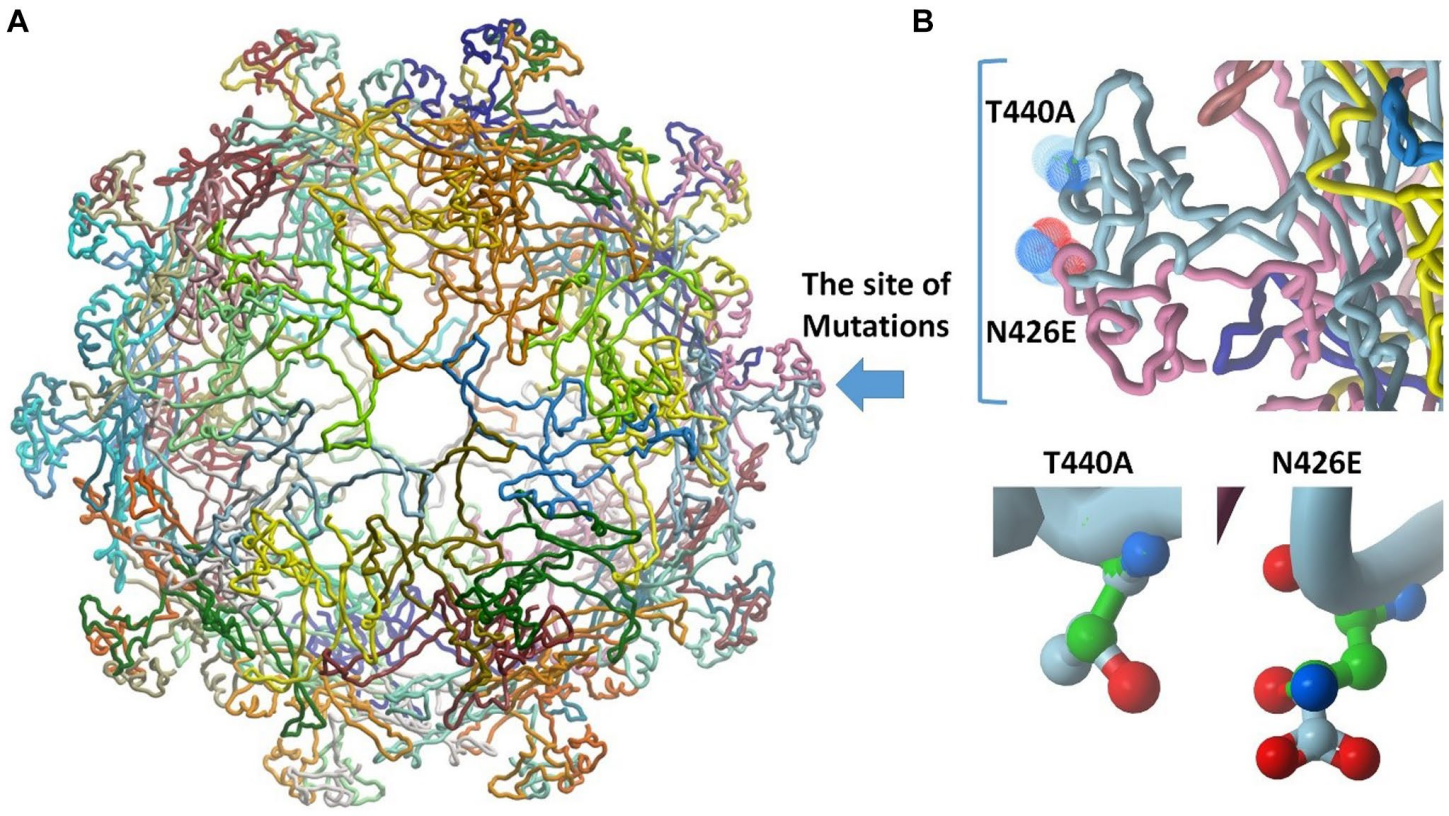
Molecular modeling studies and 3D model generation. **(A)** The obtained structure model of the retrieved sequences shows the site of mutations (blue arrow). The structure comprises 60 replicates of VP2 protein. **(B)** Insight into the site of mutation showing the N426E and T440A mutants. The backbone of alanine and asparagine are displayed in green. The structure of glutamate and threonine is colored by atoms.

The VP2 protein structures of the sequenced hits were generated using the SWISSMODEL website (Figures 5AB). All the sequences exhibited the closest resemblance to the canine parvovirus capsid VP2 protein structure with the PDB ID 1P5Y, determined at a resolution of 3.20 Å. The percentage of similarity in sequence between the reference and obtained sequences was 98.86%, with a complete coverage of 100%. The structural analysis demonstrated that neither of the mutants, N426E and T440A, have binding sites at the interface of capsid proteins; instead, they are both oriented toward the solvent (Figure 5A). For deeper understanding of the impact of mutations on the overall binding strength among VP2 protein monomer interactions, calculations were performed to assess the binding energy and the dissociation constant (Kd). Results showed that both mutations induced a decrease in the binding energy of ΔΔG = −2.026 kcal/mol and in Kd by 0.033. This indicates stronger binding of VP2 monomers in the mutant forms. This might contribute to more stabilized capsid in the mutant forms of the virus.

### 3.6 Predicted changes in antigenicity

Table 3 provides a summary of changes in the antigenicity of the canine parvovirus capsid VP2 protein across different variants. Antigenic probability values have been calculated, along with corresponding antigenic scores, for each protein. The antigenic probability values range from 0.5390 to 0.5513, indicating the likelihood of these variants to be recognized by the immune system. The antigenic scores mirror these probabilities and suggest a consistent antigenic nature among the listed variants. The “Antigenic probability” column seems to indicate the likelihood of a protein to trigger an immune response. The values for all variants fall within a relatively close range, suggesting that these proteins share similar potential for antigenicity. However, the mutants has a little bit lower antigenicity score, which might indicate that the mutants might escape the immune reaction and hold possible immune resistance.

**Table 3:** The changes in canine parvovirus capsid VP2 protein antigenicity.

Protein	Antigenic probability	Antigenic score
Wild type	Antigenic	0.5513
MW544031	Antigenic	0.5390
MW544032	Antigenic	0.5390
MW544033	Antigenic	0.5441
MW544034	Antigenic	0.5441

## 4 Discussion

Canine parvovirus is a serious infectious viral disease, which attacks puppies during the first few months of their lives. Clinical diagnosis of CPV infection seems very difficult since vomiting and diarrhea are similar to other enteric diseases (Parthiban et al., 2012).In the current study, the identification and molecular characterization of CPV-2 from rectal swabs collected from diseased dogs were done using IC, PCR and RFLP followed by VP2 sequencing and phylogenetic analysis. The infection rate was higher in dogs aged less than 6 months which is in accordance with the hypothesis that maternal antibodies are transmitted via colostrum, protecting the young puppies against infectious diseases (Sharma et al., 2018). In contrast, a previous work (Phukan et al., 2010) recorded the highest infection rate among dogs aging 6–12 months suggesting that it may be due to vaccination failure. Regarding breed disposition, the German shepherd breed was found to be the most predisposed breed in this study followed by Pit bull, Golden Pikinwa, Boxer and Griffon. This came in agreement with previous studies which reported that Pit bull and German shepherd are more susceptible to be infected with CPV-2 than other dog breeds (AL-Hosary, 2018; Elbaz et al., 2021). The immunochromatographic assay is a simple, inexpensive, and rapid CPV diagnostic method available in veterinary clinic practice. It was able to detect (264/272) (82.5%) of infected cases. Meanwhile, PCR technology seems more sensitive than IC for the detection of CPV-2, as it was able to detect 272/320 (85%) of infected cases based on the amplification of a highly conserved region of the VP2 gene, which is similar to previous research at the national level (Elbaz et al., 2021). Previous study revealed that negative IC test results do not exclude CPV-2 infection, but positive IC test result always indicates CPV-2 infection (Kantere et al., 2015). The high rate of PCR-positive samples (85%) suggests that CPV2 plays a significant role in producing diarrhea in puppies in Egypt, as previously documented (Zaghawa and Abualkhier, 2019; Sayed-Ahmed et al., 2020). In the present study, many animals were unvaccinated which enforces the need to increase vaccination efforts to decrease CPV-2 prevalence. However, CPV-2 negative results suggested that other contributing factors may be associated with severe diarrhea, which needs more investigation (Castillo et al., 2020). Mutation of the VP2 is critical in CPV evolution playing a significant role in the differentiation of CPV-2 variants. Molecular and sequence analysis of the VP2 gene of CPV-2 is the gold standard for identifying CPV-2 strains and provides crucial information on the circulating viruses in the study area and their relationship with other worldwide circulating strains (Hoang et al., 2019).

The RFLP approach with the MboII restriction enzyme was utilized successfully to differentiate CPV-2 variants (Moon et al., 2020). The nucleotide variation of strains CPV/Egy2/2021 and CPV/Egy4/2021 created an *Mbo*II restriction site (GAAGA) unique to CPV-2c, and so it was possible to distinguish these genotypes from the CPV-2a/2b by simple digestion of the 583 bp amplicon that yielded two fragments of about 500 and 80 bp in size. This is similar to a previous work (Buonavoglia et al., 2001) that found that the PCR-RFLP assay with enzyme *Mbo*II can only detect CPV-2c genotypes. Other studies revealed that the PCR-RFLP test had a 100% typeability, while that of the southern blot test was only (75%) (Smith et al., 2002). On the other hand, this work is inconsistent with previous research (Castro et al., 2011) which reported that CPV-2a can display the same RFLP results as CPV 2c, suggesting that *Mbo*II-based RFLP analysis is a defective technique. On the other hand, a previous study (Timurkan et al., 2019) showed that CPV 2a/2b are not digested with M*bo*II enzyme and consequently are indistinguishable, suggesting the importance of sequence analysis to definitively characterize these strains by indicating the amino acid residues (Timurkan et al., 2019). Therefore, to obtain precise results in the molecular typing of CPV-2 variants, future research must involve both sequencing and RFLP analysis (Castro et al., 2011).

Reviewing the previous studies, there are molecular characterization studies of CPV341 2 in Egypt. A lower prevalence of parvovirus (43% using PCR) (35% using IC test) was detected in an Egyptian study included three provinces (Cairo, Sohag and Assiut) (Abdel-Baky et al., 2023). Others (Zaher et al., 2020) also reported a lower prevalence of CPV-2 (40%) by rapid Ag Test Kit. In a study among 3,864 diseased dogs in Egypt, parvovirus infection was the major cause of diarrhea and vomiting (Rakha et al., 2015) The presence of CPV-2b has been reported by (Awad et al., 2019), while other study (AL-Hosary, 2018) revealed the close antigenic relationship of CPV-2a with Chinese serotypes suggested that this serotype may be introduced from China to Egypt. While (Awad et al., 2018) reported Parvovirus isolates which were 100% related to Portugalian isolates. Serotypes CPV-2b/2c were reported by Elbaz et al. (2021) and CPV- 2a and CPV- 2b were also reported in previous Egyptian studies (AL-Hosary, 2018; Soliman et al., 2018; El-Neshwy et al., 2019). At present (Zaghawa and Abualkhier, 2019) recorded that the three variants are circulating in dog population in Egypt. In this study, nucleotide sequencing of four selected samples showed that they belong to CPV-2 type 2a and 2c.

The exposed region that comprises amino acids 267–498 of the VP2 protein is identified as the large GH loop which shows the greatest variability among CPV variants exhibiting the main antigenic site which manages tissue tropism, host range and antigenicity of virions by interlinkage with cell transferrin receptors (TfR). Numerous studies reported that TfR have a significant role in the host cell susceptibility to CPV 2 infection. Replacement of amino acids of the 3 fold residues may also interfere with the neutralization of virus by monoclonal antibodies (Fagbohun and Omobowale, 2018). Since the appearance of the new viral strains, research about mutations of their amino acid has been ongoing (Hao et al., 2020). By continuous tracking of these point mutations, the emergence of new sublineages can be expected before significant changes in amino acids take place (Jantafong et al., 2022). These mutations aid in capsid stability, enhanced receptor-binding capability, resulting in wide host range, and increased the pathogenicity of the new viruses (Ndiana et al., 2022). Here, amino acid substitutions in the VP2 were detected at residue 426 in samples CPV/Egy2/2021 and CPV/Egy4/2021 (N426E) confirming the CPV-2c genotype. This amino acid substitution tendency of (N426E) (CPV-2c carrying a Glu) on residue 426 of VP2 was first detected in 2000 in Italy (Jantafong et al., 2022). This mutation has been also emphasized by Elbaz et al. (2021) who confirmed that the main residue to distinguish between the three strains was residue 426. Changing an asparagine (N) to glutamine (E) in a protein can have significant ramifications for its arrangement, operation, and interactions. Asparagine is an uncharged, polar amino acid featuring an amide side chain, while glutamine, also polar, and bears a negative charge because of its carboxylic acid side chain (illustrated in Figure 5B). The shift from a neutral side chain (Asn) to a negatively charged one (Glu) could introduce repulsions or attractions with adjacent residues due to electrostatic forces. This alteration might induce modifications in local structural components like α-helices and β-sheets. The difference in size between the two side chains might cause clashes or create novel chances for interactions within the protein’s three-dimensional structure. The negative charge of glutamine could establish fresh interactions or disrupt existing ones, potentially influencing the protein’s operation. Another amino acid mutation at residue 440 (T440A) in CPV-2a strains (CPV/Egy1/2021 and CPV/Egy3/2021) were also detected in this study. This residue which represents at the top of the threefold spike was also recorded as the major viral antigenic site and that high rates of this substitution are associated with the emerging of new CPV 2 variants (Capozza et al., 2023). This was similar to previous research (Timurkan et al., 2019) reported that (T440A) mutations have emerged probably as a result of antigenic drift of the viral genome leading to positive selection to improve escaping from the immune system suggesting that these modifications could lower vaccine efficacy and/or expand the pathogenicity of CPV-2 variants. This amino acid T440A mutation in CPV-2a strains has also observed in Italy (Dei Giudici et al., 2017), Taiwan (Chiang et al., 2016), and Korea (Yoon et al., 2009). While a previous study (Dei Giudici et al., 2017) has reported this mutation also in CPV-2b and CPV-2c strains. The mutation of threonine to alanine (depicted in Figure 5B) can yield varied outcomes based on the protein, its particular function, and the nearby conditions. Threonine (T), a polar amino acid, has a hydroxyl (-OH) group in its side chain. It is comparatively larger and bulkier than alanine (A), a nonpolar amino acid with a simple methyl group in its side chain. The hydroxyl group in threonine often engages in hydrogen bonding interactions. In contrast, alanine lacks a hydroxyl group and usually avoids hydrogen bonding interactions. It is commonly known for promoting helix formation due to its compact side chain. Substituting threonine (polar) with alanine (nonpolar) could lead to shifts in the local hydrophobic properties of the protein segment where the alteration happens. If threonine participated in hydrogen bonding interactions, the mutation could disturb those connections, potentially impacting the protein’s stability and folding. On the other hand, deduced amino acid alignment showed some unique mutations (S542L, H543Q, Q549H, and N557T) in the CPV-2 c serotype which were not detected in the present study.

## 5 Conclusion

A molecular survey of CPV-2 in domestic dogs was carried out in two governorates at the Delta region of Egypt. Results revealed that both CPV-2a and CPV-2c are obviously circulating in the study area. Deduced amino acid sequence analysis showed changes at residue (N426E) and residue (T440A). Continuous and periodical monitoring and molecular detection of CPV-2 variants should be further explored on a large scale investigations to determine the dynamics of the prevalent variants in contributing pathogenicity, host range, vaccine failure, and even the sensitivity of diagnostic tests. These data will facilitate early and proper diagnosis of the disease and aid in the development of different strategies for future vaccination.

## Data availability statement

The original contributions presented in the study are included in the article/supplementary material, further inquiries can be directed to the corresponding authors.

## Ethics statement

The study meets the guidelines of the Declaration of Helsinki and obtained the approval (approval number KFS-2019/09) from the Institutional Review Board of the Faculty of Veterinary Medicine, Kafrelsheikh University, Egypt. The studies were conducted in accordance with the local legislation and institutional requirements. Written informed consent was obtained from the owners for the participation of their animals in this study.

## Author contributions

AM: Conceptualization, Data curation, Formal analysis, Investigation, Supervision, Validation, Visualization, Writing – original draft, Writing – review & editing, Methodology, Project administration, Software. IE-K: Conceptualization, Data curation, Investigation, Supervision, Visualization, Writing – original draft, Project administration. ER-Á: Data curation, Formal analysis, Validation, Visualization, Writing – original draft, Writing – review & editing, Funding acquisition, Resources. EK: Conceptualization, Data curation, Investigation, Project administration, Supervision, Visualization, Writing – original draft, Formal analysis, Methodology, Software, Validation, Writing – review & editing. NA: Data curation, Formal analysis, Validation, Writing – original draft, Writing – review & editing, Conceptualization, Investigation, Methodology, Project administration, Software, Supervision. AlA: Formal analysis, Project administration, Validation, Visualization, Writing – original draft, Funding acquisition, Resources. ME-k: ___. AhA: Funding acquisition, Investigation, Resources, Validation, Writing – original draft, Writing – review & editing. EE: Conceptualization, Data curation, Formal analysis, Funding acquisition, Investigation, Resources, Supervision, Validation, Visualization, Writing – original draft, Writing – review & editing.

## Footnotes

^1^ http://www.ncbi.nlm.nih.gov/
